# Effects of UroVaxom on Urinary Tract Infection After Ureteroscopic Lithotripsy: A Multicenter Prospective Randomized Study

**DOI:** 10.3390/jcm15207938

**Published:** 2026-10-14

**Authors:** Sang Hee Lee, Kyung-Jin Oh, Ho Seok Chung, Cheol Kyu Oh, Myungchan Park, Hyun Tae Kim, Bum Soo Kim

**Affiliations:** 1 Department of Urology, Kyungpook National University Hospital, Daegu 41944, Republic of Korea; 06kiki@naver.com (S.H.L.); ht-kim1212@hanmail.net (H.T.K.); 2 Department of Urology, Chonnam National University Hospital, Chonnam National University Medical School, Gwangju 61469, Republic of Korea; exeokj@hanmail.net (K.-J.O.); hschung615@gmail.com (H.S.C.); 3 Department of Urology, Inje University Haeundae Paik Hospital, Inje University College of Medicine, Busan 48108, Republic of Korea; ckohuro@gmail.com (C.K.O.); mcpark@paik.ac.kr (M.P.); 4 Department of Urology, School of Medicine, Kyungpook National University, Daegu 41566, Republic of Korea

**Keywords:** ureteroscopy, lithotripsy, urinary tract infections, bacterial vaccines, urolithiasis

## Abstract

**Background/Objectives**: To evaluate whether perioperative UroVaxom reduces postoperative urinary tract infection-related findings after ureteroscopic lithotripsy. **Methods**: In this prospective, randomized, multicenter study, patients with a history of recurrent UTI who were scheduled to undergo ureteroscopic lithotripsy for unilateral renal or ureteral stones < 2 cm were assigned to UroVaxom 60 mg or placebo once daily for 1 week before and 2 weeks after surgery. Baseline characteristics, surgical outcomes, postoperative UTI, pyuria, bacteriuria, serum C-reactive protein (CRP), and adverse events were compared. **Results**: Of 73 screened patients, 57 were analyzed (placebo, *n* = 29; UroVaxom, *n* = 28). Baseline characteristics were comparable between groups. Operative time, hospital stay, and stone-free rate did not differ significantly. Febrile UTI occurred in 1 patient (3.4%) in the placebo group and in none of the UroVaxom group. At 1 month, pyuria and bacteriuria were significantly less frequent in the UroVaxom group, and mean serum CRP was also significantly lower. Adverse events were uncommon and similar between groups. **Conclusions**: Although this study did not demonstrate that perioperative UroVaxom significantly reduced the incidence of postoperative febrile urinary tract infection compared with placebo after ureteroscopic lithotripsy, UroVaxom was associated with lower rates of postoperative pyuria and bacteriuria and lower serum CRP levels at 1 month without increasing adverse events.

## 1. Introduction

Urinary tract infections (UTIs) are among the most frequent and clinically important complications after endourological procedures, including ureteroscopic lithotripsy for stone disease [1,2,3,4]. Although ureteroscopy is generally considered safe and minimally invasive, post-procedural infectious events range from transient febrile UTI to severe urosepsis, and their occurrence can substantially worsen postoperative outcomes [5]. These infections are clinically significant because they are associated with increased morbidity, prolonged hospitalization, delayed recovery, and higher healthcare costs. In addition, infectious complications after ureteroscopy often lead to escalated antibiotic exposure, which can contribute to antimicrobial resistance and undermine perioperative antimicrobial stewardship efforts. Therefore, improving infection prevention strategies in patients undergoing ureteroscopic lithotripsy remains a key priority in contemporary stone surgery [6,7].

Multiple factors contribute to infection risk in the perioperative setting. Urinary tract instrumentation itself can facilitate bacterial inoculation and biofilm formation, while pre-existing bacteriuria, even if asymptomatic, may increase the likelihood of postoperative infection after ureteroscopic intervention [2,4]. Patient- and procedure-related predictors of postoperative UTIs and urosepsis have been described, emphasizing the need for accurate risk stratification and tailored prophylactic approaches [3,4]. In stone surgery, the persistence of residual fragments after ureteroscopy may further heighten infectious risk by serving as a nidus for bacterial persistence and recurrent inflammation, particularly when stone clearance is incomplete [8]. Given these mechanisms, effective preventive strategies should not only minimize immediate postoperative infection but also reduce downstream recurrence, antibiotic use, and readmissions [6,7].

UroVaxom, an oral immunostimulant derived from an *Escherichia coli* lysate, has gained attention as a non-antibiotic prophylactic option for recurrent UTIs [9,10,11]. Its proposed mechanism involves modulation of the host immune response, including activation of innate and adaptive pathways that enhance mucosal defense against uropathogenic bacteria [12,13]. Experimental and translational studies suggest that UroVaxom can stimulate immune effector cells and antigen-presenting pathways relevant to urogenital immunity, thereby potentially improving resistance to recurrent bacterial exposure [12,13]. In clinical settings, immunoactive prophylaxis with UroVaxom has been evaluated across diverse adult populations with recurrent uncomplicated UTIs, and systematic reviews have supported its preventive benefit with acceptable safety profiles [9,10,11,14]. Moreover, clinical studies in women with recurrent cystitis and broader adult cohorts have reported reductions in UTI recurrence following UroVaxom administration, reinforcing its role as a potential adjunct or alternative to prolonged antibiotic prophylaxis [15,16,17,18].

This therapeutic concept may be particularly relevant to patients undergoing ureteroscopic lithotripsy, in whom infectious complications can be triggered by perioperative manipulation of the urinary tract and bacterial translocation during stone fragmentation [4,5]. In this context, we hypothesized that UroVaxom may provide additional protection beyond conventional antibiotic prophylaxis by enhancing host immunity during the vulnerable perioperative period, potentially mitigating postoperative bacteriuria and symptomatic infection. Recent evidence has also explored postoperative UroVaxom administration in stone-related infectious contexts, suggesting possible benefits in reducing bacteriuria associated with suspected infectious stones [19]. If UroVaxom can meaningfully reduce post-ureteroscopic infectious events, the clinical implications would extend beyond fewer UTIs to include decreased antibiotic consumption, improved antimicrobial stewardship, and faster postoperative recovery, ultimately reducing the overall burden of care [6,7].

Despite substantial evidence supporting UroVaxom in recurrent uncomplicated UTI prevention, important limitations remain when translating these findings to the postoperative ureteroscopy population. Much of the existing literature has focused on non-surgical recurrent UTIs, often in female patients, with comparatively fewer studies addressing perioperative infectious outcomes following endourological interventions [9,10,11,14,15,16,17,18,20]. In addition, large-scale, multicenter randomized controlled trials specifically evaluating UroVaxom for prevention of post-ureteroscopic lithotripsy infections are lacking, and current evidence in stone surgery is limited to narrower clinical contexts rather than standardized post-URS infectious endpoints [19]. Given the established predictors of infection after ureteroscopy and the ongoing emphasis on antimicrobial prophylaxis and stewardship in urologic surgery, this represents a meaningful knowledge gap with direct clinical relevance [3,4,6,7]. Accordingly, the present study was designed to evaluate the clinical utility of UroVaxom in preventing infectious complications in patients undergoing ureteroscopic lithotripsy.

## 2. Materials and Methods

### 2.1. Research Ethics

This study was performed according to the ethical standards of the 1964 Declaration of Helsinki and its later amendments. Patient enrollment, data collection, and analysis were performed after approval by the Institutional Review Board (KNUH 2021-09-025).

### 2.2. Trial Registration

This study was registered in the Clinical Research Information Service (CRIS) under the title “Efficacy of Urovaxom to prevent postoperative febrile urinary tract infection for ureteroscopic ureterolithotomy in patients with previous urinary tract infection: prospective randomized study” (registration number: KCT0006991; registration date: 10 February 2022; URL: https://cris.nih.go.kr/cris/search/detailSearch.do?seq=19984&search_page=L, accessed on 1 September 2026).

### 2.3. Patients and Methods

This prospective, randomized, multicenter, parallel-group intervention study was conducted to evaluate the preventive effect of UroVaxom on urinary tract infections (UTIs) following ureteroscopic lithotripsy. The study was designed as a prevention trial comparing perioperative UroVaxom with placebo. The off-label perioperative use of UroVaxom was reviewed and approved by the Institutional Review Board. No significant changes to the trial methods, outcomes, or analyses were made after the start of the clinical trial.

Patients with a history of recurrent UTI who were scheduled to undergo ureteroscopic lithotripsy for unilateral ureteral or renal stones < 2 cm were considered for enrollment between October 2021 and December 2024. Patients were recruited from six university hospitals located in Daegu, Busan, Gwangju, and Jeonnam, South Korea. These hospitals were selected because stone surgery could be performed by experienced surgeons at the level of assistant professor or higher. Recurrent UTI was defined as ≥2 episodes of UTI within 6 months or ≥3 episodes within 12 months.

Patients were excluded if they had an untreated urinary tract infection, bilateral stones, a preoperatively indwelling ureteral stent or nephrostomy catheter, immunosuppression or immunodeficiency, pregnancy, concomitant anatomical abnormalities of the urinary tract such as ureteral stricture, or a prior history of adverse events or hypersensitivity related to UroVaxom.

The original sample-size calculation was based on a non-inferiority framework for postoperative febrile UTI. Based on previous studies in comparable populations, the anticipated infection rates were 10% in the control group and 5% in the UroVaxom group, with a prespecified absolute non-inferiority margin of 5 percentage points. Using a normal approximation for two independent proportions with equal allocation, a one-sided α level of 0.05, and 80% power, approximately 85.6 evaluable patients were required per group, rounded up to 86. Allowing for an anticipated dropout rate of 10%, the planned recruitment target was 96 patients per group, for a total of 192 patients.

Eligible patients were randomly assigned in a 1:1 ratio to the UroVaxom group or placebo group using a computer-generated randomization sequence. The randomization sequence was generated by the corresponding author. Personnel involved in patient enrollment and treatment allocation did not have access to the random allocation sequence. Allocation was concealed until enrollment. This was a single-blind trial in which the study participants were blinded to treatment allocation. The placebo was identical in appearance to UroVaxom.

After providing informed consent, participants assigned to the UroVaxom group received UroVaxom 60 mg, containing 6 mg of standardized lyophilized *Escherichia coli* bacterial lysate, once daily. Participants assigned to the control group received a matching placebo according to the same schedule. Treatment was started 1 week before surgery and continued for 2 weeks postoperatively.

The duration of administration was determined with consideration of real-world clinical practice. In patients with ureteral stones, prolonged delay between diagnosis and surgery is often not feasible. Therefore, the preoperative treatment period was set at 1 week, taking into account the time required for preoperative evaluation and surgical scheduling. The postoperative treatment period was set at 2 weeks, corresponding to the routine timing of double-J stent removal.

All patients underwent standard ureteroscopic lithotripsy for stone removal. Following the procedure, a double-J stent was placed in each patient and subsequently removed 2 weeks after surgery. Baseline characteristics, including age, sex, comorbidities, stone size, and stone location, were recorded for all participants. Surgical outcomes, including operative time, hospital stay, perioperative complications, and stone-free rate, were assessed. Stone-free status was evaluated at 1 month postoperatively using non-contrast computed tomography and was defined as no visible residual fragment.

The primary endpoint was the incidence of postoperative febrile UTI. Secondary endpoints included postoperative pyuria, postoperative bacteriuria, serum C-reactive protein (CRP) levels, operative time, hospital stay, stone-free rate, perioperative complications, and drug-related adverse events. Postoperative outcomes, including the incidence of UTI, pyuria, and bacteriuria, were evaluated within 24 h and at 1 month after surgery. Serum CRP levels were measured at the same time points to assess postoperative inflammation. Adverse events related to the intervention were monitored throughout the study period.

Pyuria was defined as >5 white blood cells per high-power field. Preoperative urinalysis was performed within 30 days before surgery. Bacteriuria was defined as bacterial growth of ≥10^5^ colony-forming units/mL in a urine culture obtained at each assessment time point. All patients had negative preoperative bacteriuria and urine culture results. Postoperative febrile UTI was defined as fever of 38 °C or higher with pyuria or bacteriuria in the absence of another infectious source. Postoperative bacteriuria without fever was classified as asymptomatic bacteriuria.

### 2.4. Statistical Analysis

The primary endpoint was the incidence of postoperative febrile urinary tract infection (UTI). Given the substantially lower-than-planned enrollment, the study was interpreted as an exploratory randomized trial, and no claim of non-inferiority was made. The incidence of febrile UTI was summarized as the number and percentage of patients in each group. The between-group risk difference (UroVaxom minus placebo) and its 95% confidence interval were estimated using an appropriate method for comparing two independent proportions, accounting for the sparse event data. The estimated risk difference and confidence interval were interpreted in relation to the prespecified non-inferiority margin of 5 percentage points.

Secondary outcomes were analyzed using appropriate methods according to the type and distribution of the variables. Continuous variables were summarized as means and standard deviations or medians and interquartile ranges, as appropriate, and categorical variables as counts and percentages. Between-group comparisons were performed using Student’s *t*-test or a suitable nonparametric test for continuous variables and the chi-square test or Fisher’s exact test for categorical variables, as appropriate. Secondary outcome analyses were considered exploratory, and their interpretation took into account the limited sample size and the potential for multiplicity. No post hoc power calculation was performed. All statistical tests for secondary outcomes were two-sided, with a nominal significance level of 0.05. Statistical analyses were performed using IBM SPSS Statistics 19.0, supplemented by an appropriate statistical procedure for estimating the risk difference and its confidence interval.

## 3. Results

### 3.1. Study Design and Participants

**Figure 1:**
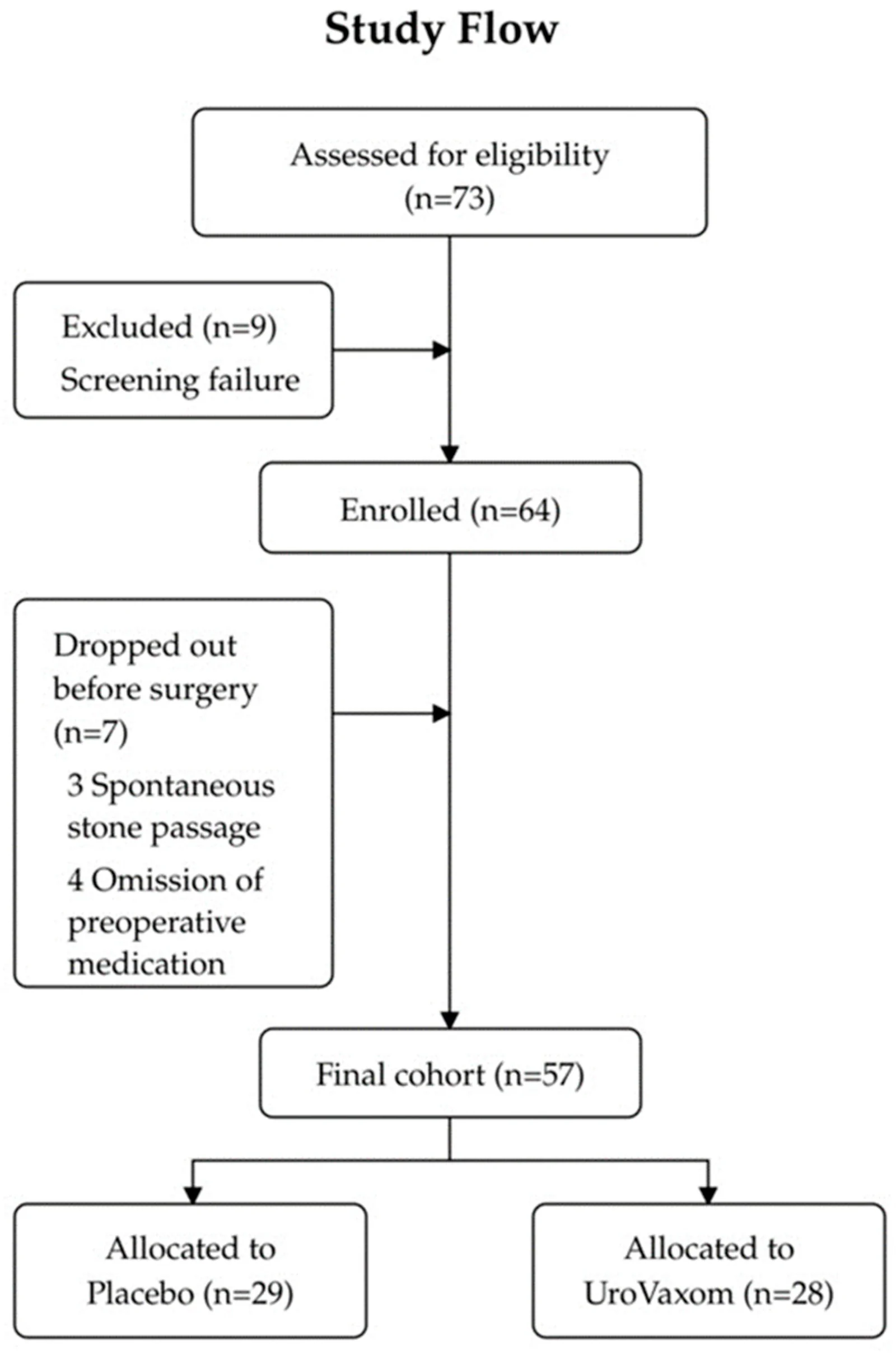
Participant Enrollment and Study Flow.

Among the six participating hospitals, four actively enrolled patients, and a total of 73 patients were registered from October 2021 to December 2024. The intervention was generally delivered as intended. However, 9 patients were excluded because of screening failure, and 7 dropped out (3 because of spontaneous stone passage and 4 because of omission of preoperative medication). After excluding patients who did not complete the study or were not eligible for final analysis, 57 patients were included in the final analysis, comprising 29 patients in the control group and 28 patients in the UroVaxom group (Figure 1). Recruitment was slower than anticipated because of the restrictive eligibility criteria and the limited number of eligible patients. The recruitment period was extended twice to facilitate attainment of the planned sample size. Despite these extensions, further recruitment became impracticable.

### 3.2. Patients Characteristics

Baseline demographic and clinical characteristics were generally comparable and balanced between the placebo group (n = 29) and the UroVaxom group (n = 28) (Table 1). The mean age was 51.6 years in the placebo group and 61.1 years in the UroVaxom group, with a comparable sex distribution (13/16 vs. 18/10, male/female). Mean body mass index was similar between the two groups (24.1 vs. 26.5 kg/m^2^). Comorbidity profiles were also comparable, including hypertension (41.4% vs. 39.3%), diabetes mellitus (31.0% vs. 21.4%), cerebrovascular disease (6.9% vs. 0.0%), heart disease (13.8% vs. 10.7%), gout (6.9% vs. 0.0%), and thyroid disease (17.2% vs. 7.1%). Stone-related baseline variables were likewise similar between the two groups. Stone laterality was comparable (right/left, 17/12 vs. 18/10), and stone location did not differ materially (kidney: 41.4% vs. 32.1%; upper ureter: 37.9% vs. 32.1%; middle ureter: 17.2% vs. 21.4%; lower ureter: 3.4% vs. 14.3%). Mean stone size was 11.0 mm in the placebo group and 10.0 mm in the UroVaxom group, and mean stone attenuation was 962.5 vs. 703.5 Hounsfield units. Radiopaque stones predominated in both groups (89.7% vs. 92.9%). Baseline infection-related parameters were also similar, including preoperative pyuria (89.7% vs. 82.1%) and baseline CRP (0.61 vs. 0.47 mg/dL).

### 3.3. Perioperative and Surgical Outcomes

Perioperative and surgical outcomes were comparable between the placebo group and the UroVaxom group (Table 2). Mean operative time did not differ between groups (54.7 vs. 58.4 min; *p* = 0.798), and mean length of hospital stay was similar (3.5 vs. 3.7 days; *p* = 0.564). Stone-free rate was also comparable (82.8% vs. 78.6%; *p* = 0.474), suggesting that UroVaxom administration did not adversely affect procedural performance or immediate surgical efficacy. Safety outcomes were favorable. No procedure-related complications were observed in either group, and overall tolerability was good. A single drug-related adverse event occurred in the UroVaxom group (3.6%), described as finger skin desquamation, with no apparent increase in other adverse events compared with placebo (*p* = 0.491).

### 3.4. Postoperative Infectious Outcomes

Postoperative febrile UTI occurred in 1 of 29 patients (3.4%) in the placebo group and in none of the 28 patients (0%) in the UroVaxom group (Table 3). The estimated risk difference (UroVaxom minus placebo) was −3.4 percentage points, with a 95% confidence interval of approximately −17.2 to 8.9 percentage points using the Newcombe–Wilson method. The upper confidence limit exceeded the prespecified non-inferiority margin of 5 percentage points. Therefore, non-inferiority could not be established. The low number of primary endpoint events and the limited sample size substantially restricted the precision of the treatment effect estimate. However, UroVaxom was associated with significantly lower rates of objective inflammatory and microbiologic findings. Postoperative pyuria assessed within 24 h of surgery was comparable (69.0% vs. 64.3%; *p* = 0.463), whereas at 1 month the proportion of patients with pyuria was significantly lower in the UroVaxom group (51.7% vs. 42.9%; *p* = 0.034). Similarly, postoperative bacteriuria was significantly less frequent in the UroVaxom group both within 24 h (17.2% vs. 3.6%; *p* = 0.015) and at 1 month (34.4% vs. 21.4%; *p* = 0.021). Serum CRP was similar within 24 h postoperatively (0.39 vs. 0.50; *p* = 0.760) but was significantly lower at 1 month in the UroVaxom group (4.27 vs. 0.13; *p* = 0.008).

## 4. Discussion

This prospective, randomized, multicenter study investigated whether perioperative UroVaxom influences postoperative infectious outcomes and inflammatory activity in patients undergoing ureteroscopic lithotripsy for unilateral renal or ureteral stones. Infectious complications following ureteroscopy remain clinically relevant, ranging from febrile UTI to urosepsis, and are associated with increased morbidity, prolonged hospitalization, and greater healthcare utilization [1,2,3,4,5]. In addition to patient- and procedure-related factors, urinary tract instrumentation itself and residual fragments may contribute to microbial persistence and postoperative inflammatory burden. Against this background, identifying adjunctive, non-antibiotic preventive strategies that can reduce postoperative urinary inflammation while supporting antimicrobial stewardship is of increasing interest [6,7].

One point that warrants consideration is the high prevalence of preoperative pyuria observed in both groups. In this study, pyuria was defined as >5 white blood cells per high-power field, and preoperative urinalysis was performed within 30 days before surgery. Importantly, all patients had negative preoperative urine culture results; therefore, the high rate of preoperative pyuria should not be interpreted as evidence of active urinary tract infection [2,3,4]. Rather, it is more likely to reflect sterile inflammatory changes associated with urinary stone disease, including urothelial irritation, urinary obstruction, hydronephrosis, prior urinary tract instrumentation, or persistent inflammatory responses related to previous urinary tract infection [4,5]. This distinction is important when interpreting the postoperative findings of the present study, because it suggests that pyuria in this setting may represent baseline stone-related inflammation as well as infection-related change.

In the present study, UroVaxom did not compromise procedural performance or early surgical efficacy. Operative time, length of hospital stay, and stone-free rate were comparable between the placebo and UroVaxom groups, indicating that UroVaxom administration did not adversely affect perioperative management or stone clearance. Clinically overt UTI during the postoperative period was infrequent in both groups and did not differ statistically, although the event rate was numerically lower in the UroVaxom group. Importantly, UroVaxom use was associated with significantly lower rates of postoperative pyuria and bacteriuria at 1 month, as well as a significantly lower serum CRP level, suggesting attenuation of postoperative urinary tract inflammation and subclinical microbial burden. From a clinical perspective, these findings are notable because pyuria and bacteriuria after ureteroscopic manipulation may reflect ongoing urothelial irritation, bacterial colonization, or low-grade infection even when formal UTI criteria are not met [4,5]. Thus, improvements in microbiologic and inflammatory markers may represent a meaningful intermediate benefit in stewardship-oriented perioperative care [6,7].

The biological plausibility of these observations is supported by established immunomodulatory effects of UroVaxom. UroVaxom is a lyophilized *Escherichia coli* lysate designed to enhance host defense against uropathogens through stimulation of innate and adaptive immune responses [9,10,11]. Experimental work has shown immunogenicity after oral administration and activation of immune effector pathways relevant to mucosal defense [11,12,21,22]. Mechanistic data suggest that UroVaxom can modulate leukocyte function, enhance cytokine production, and increase natural killer activity [12,22]. In addition, UroVaxom has been shown to activate dendritic cells, supporting antigen presentation and downstream adaptive responses that may be important for sustained mucosal protection in the urinary tract [13]. Contemporary discussions on persistent bacterial cystitis and vaccine development also support the concept that targeting host–pathogen interactions and mucosal immunity may influence bacterial persistence and inflammatory activity beyond what antibiotics alone can achieve [23].

Patient selection and the causative uropathogen should also be considered when interpreting these findings. UroVaxom is a bacterial lysate derived from 18 *Escherichia coli* strains, and experimental studies have demonstrated effects on innate and adaptive immune responses, including leukocyte and dendritic-cell activation [12,13]. Although these findings provide a biological rationale for immunoprophylaxis, they do not establish clinical protection against non-E. coli uropathogens in the perioperative setting. The present study enrolled patients with a history of recurrent UTI without restricting eligibility to documented recurrent E. coli infections, and treatment effects were not evaluated according to the causative organism. Consequently, pathogen-specific efficacy cannot be inferred from our results. Given the selected study population and the absence of a demonstrated reduction in postoperative febrile UTI, these findings do not support routine UroVaxom administration to all patients undergoing ureteroscopic lithotripsy. Further studies incorporating microbiological characterization and pathogen-specific analyses are needed to identify patients most likely to benefit.

Our results are generally consistent with prior clinical evidence supporting UroVaxom for recurrent UTI prevention. Randomized and observational studies have reported reductions in recurrence frequency and favorable tolerability in adult populations, particularly among patients with recurrent cystitis [15,16,17,18]. Systematic reviews have likewise concluded that immunoactive prophylaxis with UroVaxom can reduce UTI recurrence with an acceptable safety profile [9,10,11,14]. A key clinical motivation for non-antibiotic prophylaxis is the potential to reduce antibiotic exposure while maintaining infection control, aligning with stewardship priorities in urologic practice [6,24]. Although most data derive from non-surgical recurrent UTI cohorts, emerging evidence has begun to evaluate postoperative UroVaxom in stone-related infectious settings, where reductions in bacteriuria have been reported [19].

At the same time, evidence across immunostimulant and vaccine-based strategies remains heterogeneous, and null or mixed results have been described. In neurogenic bladder dysfunction, a pilot randomized trial did not demonstrate a clear reduction in UTI frequency with immunotherapy [25]. Reviews of non-antibiotic approaches emphasize variability in study design, patient selection, and outcome ascertainment, which can contribute to inconsistent effect estimates across interventions and settings [26,27]. Even within urologic oncology, where UroVaxom has been explored to reduce infectious adverse effects during intravesical therapy, benefits may depend on context-specific mechanisms and endpoints [28]. Thus, while the aggregate literature supports UroVaxom for recurrent uncomplicated UTI prevention [9,10,11,14], translation to post-procedural infections remains less definitive, reinforcing the value of procedure-specific trials. While a 3-month course of UroVaxom is commonly recommended for prevention of recurrent UTI, no established guideline currently exists regarding the optimal duration of administration for prevention of procedure-related UTI. Therefore, in this study, the treatment duration was pragmatically determined as 1 week before surgery and 2 weeks after surgery, taking into account the usual interval between stone diagnosis and surgery, the minimum anticipated time to drug effect, and the postoperative indwelling period of the double-J stent. Further studies are needed to define the optimal duration of UroVaxom administration in this setting.

Several considerations may explain why reductions were observed in pyuria, bacteriuria, and CRP but not in symptomatic UTI. First, postoperative symptomatic UTI after ureteroscopy-particularly within a short follow-up window-may be a relatively low-frequency outcome in well-managed cohorts, limiting statistical power in modestly sized trials [1,2,3,4,5]. Second, symptom-based UTI definitions are sensitive to ascertainment and may be influenced by postoperative irritative voiding symptoms, stent-related discomfort, and variable thresholds for obtaining cultures or prescribing antibiotics [4,5]. In contrast, pyuria and bacteriuria may be more common and may capture subclinical bacterial persistence or inflammation that does not always progress to symptomatic infection. Third, UroVaxom may exert its principal benefit by reducing colonization and inflammatory tone rather than preventing all symptomatic events, particularly when standard perioperative antibiotic prophylaxis is already applied, and baseline infection risk is controlled [6,7].

This study addresses a clinically relevant gap by evaluating UroVaxom specifically in the context of ureteroscopic lithotripsy, where instrumentation-related microbial translocation and postoperative inflammatory sequelae are well recognized [4,5]. Strengths of this study include its prospective randomized design, multicenter participation, and concurrent assessment of microbiologic and inflammatory endpoints. Nevertheless, several limitations should be acknowledged. Incomplete recruitment represents a major limitation of this study. In Korea, UroVaxom is approved for recurrent UTI, and we restricted enrollment to patients with recurrent UTI who were scheduled to undergo ureteroscopic lithotripsy. Although this restriction ensured a clinically relevant study population, it limited the number of eligible patients. The study was terminated before reaching the planned sample size because of recruitment difficulties. Consequently, the precision of the estimated treatment effect was substantially limited, particularly for the primary endpoint, for which only one febrile UTI event was observed. Although the observed incidence of febrile UTI was numerically lower in the UroVaxom group, the confidence interval for the between-group risk difference extended beyond the prespecified non-inferiority margin. Accordingly, the present findings do not establish non-inferiority of UroVaxom to placebo. The absence of a statistically significant difference in the primary endpoint should not be interpreted as evidence of equivalence or non-inferiority. The findings for secondary endpoints should likewise be considered exploratory and require confirmation in adequately powered randomized trials.

Additional limitations include the short follow-up period, which precluded assessment of longer-term recurrence, delayed infections, or sustained immunologic effects. Moreover, although antimicrobial stewardship is a central rationale for immunoprophylaxis, quantitative antibiotic consumption and resistance patterns were not captured, and patient-centered outcomes were not evaluated. Future large-scale prospective studies with longer follow-up, standardized infection definitions, and broader endpoints including antibiotic exposure, resistance ecology, and patient-reported outcomes are needed to better define the clinical role of UroVaxom in perioperative stone surgery [6,24,26,27,29].

## 5. Conclusions

This study did not demonstrate that perioperative UroVaxom significantly reduced the incidence of postoperative febrile urinary tract infection compared with placebo after ureteroscopic lithotripsy. However, UroVaxom was associated with lower rates of postoperative pyuria and bacteriuria, as well as lower serum CRP levels at 1 month, suggesting a possible adjunctive role in reducing postoperative urinary inflammatory and microbiologic findings.

## Figures and Tables

**Table 1:** Baseline Demographic and Clinical Characteristics.

	Control (n = 29)	UroVaxom (n = 28)
Mean age (years)	51.6	61.1
Male/female	13/16	18/10
Mean BMI (kg/m^2^)	24.1	26.5
Hypertension	12 (41.4%)	11 (39.3%)
Diabetes mellitus	9 (31.0%)	6 (21.4%)
Cerebrovascular disease	2 (6.9%)	0 (0.0%)
Heart disease	4 (13.8%)	3 (10.7%)
Gout	2 (6.9%)	0 (0.0%)
Thyroid disease	5 (17.2%)	2 (7.1%)
Stone position Kidney Upper ureter Middle ureter Lower ureter	12 (41.4%) 11 (37.9%) 5 (17.2%) 1 (3.4%)	9 (32.1%) 9 (32.1%) 6 (21.4%) 4 (14.3%)
Stone laterality (right/left)	17/12	18/10
Mean stone size (mm)	11.0	10.0
Mean stone attenuation (HU)	962.5	703.5
Radiopaque stone	26 (89.7%)	26 (92.9%)
Preoperative pyuria	26 (89.7%)	23 (82.1%)
Mean preoperative CRP (mg/dL)	0.61	0.47

Values are presented as mean or number (%). Percentages may not total 100% because of rounding. Patients were randomly assigned in a 1:1 ratio to the UroVaxom or placebo group using a computer-generated randomization sequence.

**Table 2:** Perioperative and Surgical Outcomes.

	Control (n = 29)	UroVaxom (n = 28)	*p*-Value
Mean operative time (min)	54.7	58.4	0.798 ^(a)^
Mean hospital stay (d)	3.5	3.7	0.564 ^(a)^
Stone-free	24 (82.8%)	22 (78.6%)	0.474 ^(b)^
Other complication	0 (0.0%)	0 (0.0%)	1.000 ^(c)^
Drug-related adverse event	0 (0.0%)	1 (3.6%) *	0.491 ^(c)^

* Finger skin desquamation. Values are presented as mean or number (%). ^(a)^ Student’s *t*-test; ^(b)^ Chi-square test; ^(c)^ Fisher’s exact test.

**Table 3:** Postoperative Infectious Outcomes.

	Control (n = 29)	UroVaxom (n = 28)	*p*-Value
Febrile UTI	1 (3.4%)	0 (0.0%)	0.509 ^(c)^
Postoperative pyuria			
POD < 24 h	20 (69.0%)	18 (64.3%)	0.463 ^(b)^
POD 1 month	15 (51.7%)	12 (42.9%)	0.034 ^(b)^
Postoperative bacteriuria			
POD < 24 h	5 (17.2%)	1 (3.6%)	0.015 ^(c)^
POD 1 month	10 (34.5%)	6 (21.4%)	0.021 ^(b)^
Postoperative CRP (mg/dL)			
POD < 24 h	0.39	0.50	0.760 ^(a)^
POD 1 month	4.27	0.13	0.008 ^(a)^

Values are presented as mean or number (%). Statistically significant at *p* < 0.05. ^(a)^ Student’s *t*-test; ^(b)^ Chi-square test; ^(c)^ Fisher’s exact test.

## Data Availability

The original contributions presented in this study are included in the article. Further inquiries can be directed to the corresponding author.

## Abbreviations

The following abbreviations are used in this manuscript:

BMI	Body mass index
CRP	C-reactive protein
HU	Hounsfield unit
POD	Postoperative day
URS	Ureteroscopy
UTI	Urinary tract infection
